# Supplementary material for: Constraints to gene flow increase the risk of genome erosion in the Ngorongoro Crater lion population
Source: Commun Biol. 2025 Apr 21;8:640. doi: 10.1038/s42003-025-07986-0 (PMC12012037; doi:10.1038/s42003-025-07986-0)
Supplement: Supplementary file 2 — Supplementary Information [file 42003_2025_7986_MOESM2_ESM.pdf]

Supplementary Information

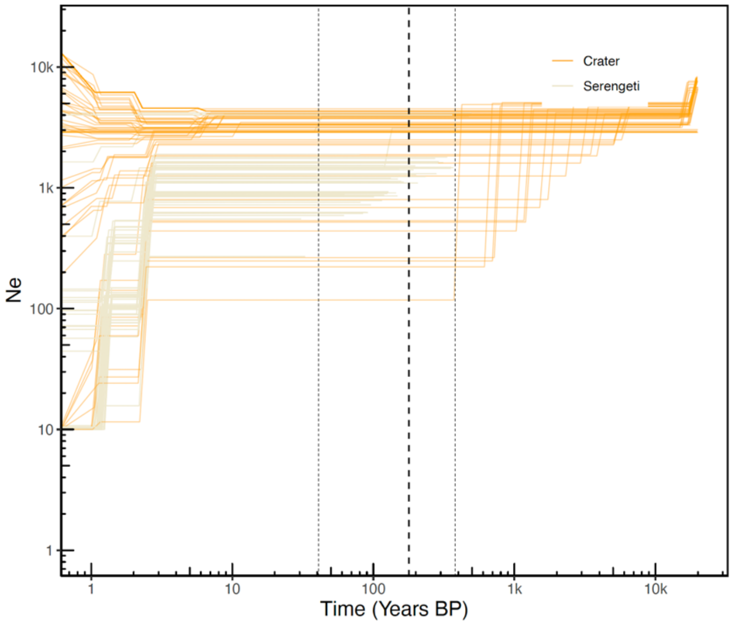

Supplementary Figure 1. Recent divergence between the Ngorongoro Crater and Serengeti plains (Ndutu, n=2; Endulen, n=1; Tanzania, n=2) using the SMC++ and 50 bootstrap runs. Dashed and dotted lines depict the mean 95% CI, respectively. Mean: 178.7179; 95% HPD: 41- 379.8.

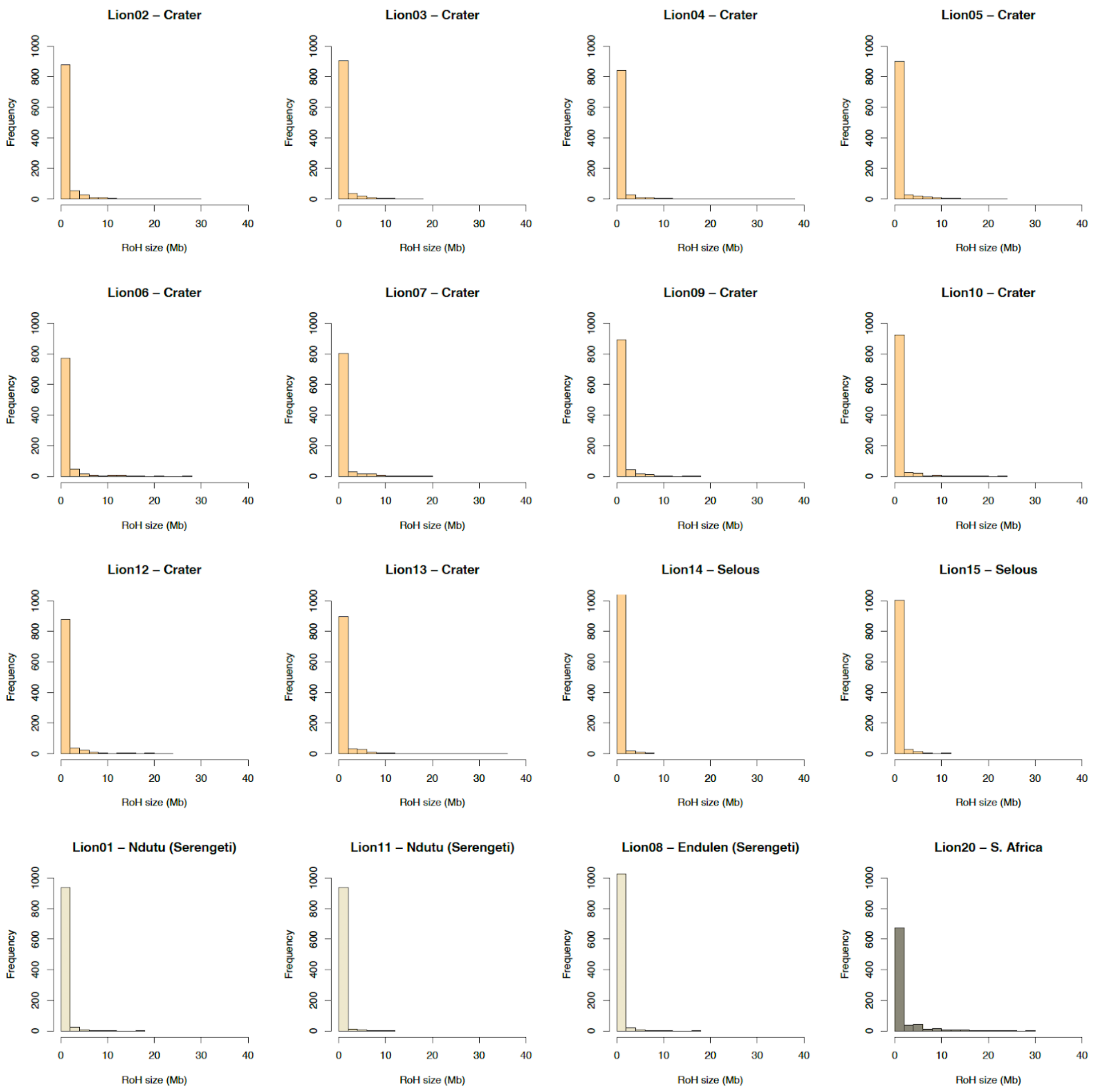

Supplementary Figure 2. ROH length distribution per individual genome. The length of those ROH is used to infer the approximate timing of inbreeding events (see Table S5).

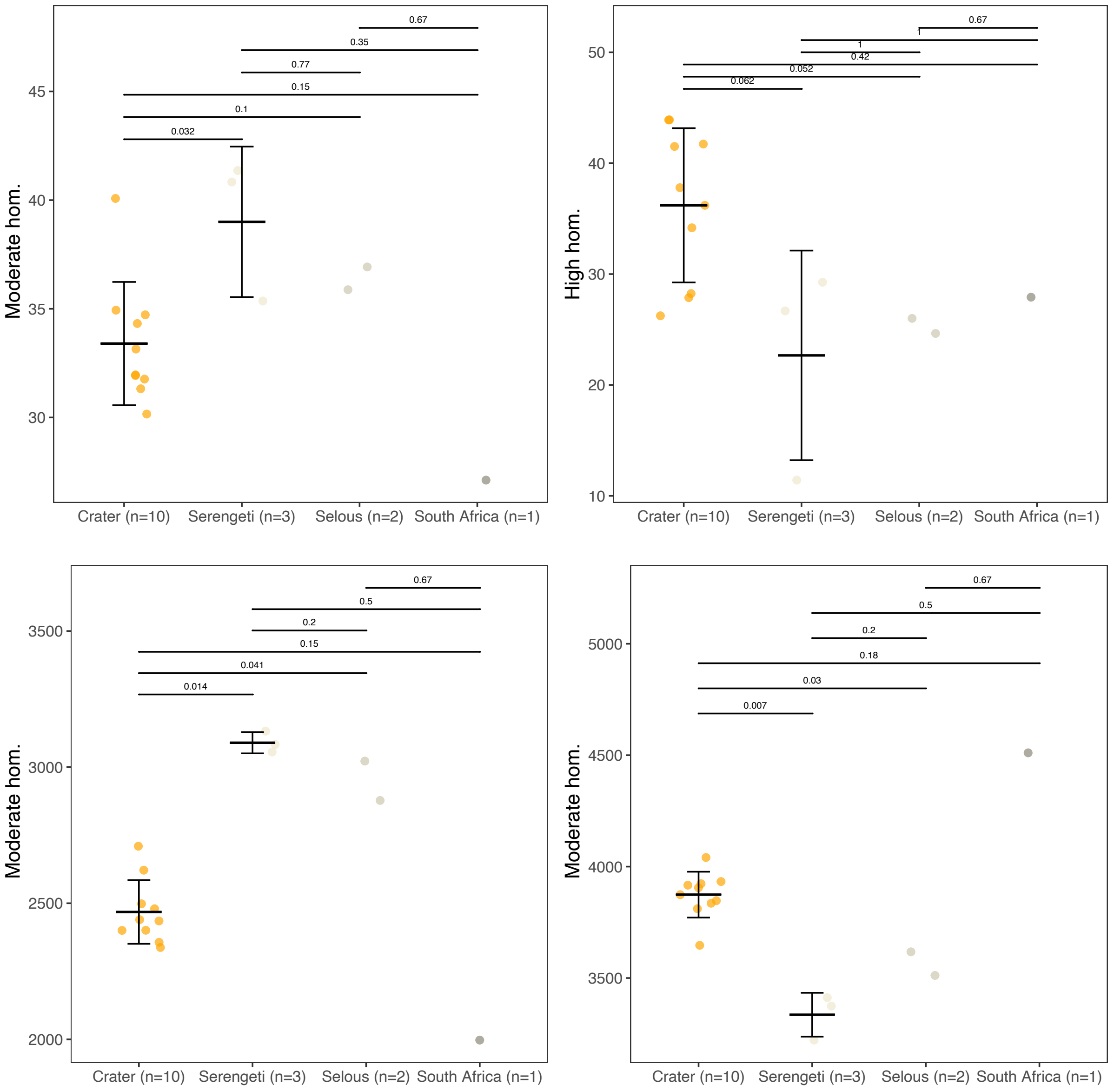

Supplementary Figure 3. Counts of High and Moderate impact variants separated by heterozygous or homozygous state for 16 lions (with sequencing depth 14X). Horizontal lines within boxplots depict the mean, bounds of boxes represent the standard deviation, and vertical bars represent minima and maxima. Statistical significance was assessed with Wilcoxon signed-rank test (n=4).
