# Supplementary material for: Constraints to gene flow increase the risk of genome erosion in the Ngorongoro Crater lion population
Source: Commun Biol. 2025 Apr 21;8:640. doi: 10.1038/s42003-025-07986-0 (PMC12012037; doi:10.1038/s42003-025-07986-0)
Supplement: Supplementary file 3 — Description of Additional Supplementary Files [file 42003_2025_7986_MOESM3_ESM.docx]

Description of Additional Supplementary Files

**File Name:** Supplementary Data 1-8

**Descriptions:**

- Supplementary Data 1: Sample metadata including newly-sequenced and public data.
- Supplementary Data 2: Ne Estimates using GONE for 10 replicates.
- Supplementary Data 3: Demographic data for the Ngorongoro Crater population. Related to Figure 2c.
- Supplementary Data 4: Summary table for heterozygosity, inbreeding (FROH) and number of variants identified with SNPeff in heterozygous or homozygous state.
- Supplementary Data 5: Timing of inbreeding events based on ROH lengths assuming a generation time of 5 years and the Panthera sp. recombination rate19. Large ROH indicate recent inbreeding events and smaller ROH, older ones. If a ROH is ≥ 40 Mb, it indicates that the inbreeding event occurred within the past 3 years ago but not before then. Conversely, if a ROH is < 0.1, it means that the inbreeding event is older than 2250 years.
- Supplementary Data 6: Allele frequencies of deleterious variants identified with SNPeff.
- Supplementary Data 7: Jax database output table for genes carrying high impact variants.
- Supplementary Data 8: Demographic and genomic parameters used in the SLiM simulations.
