# Supplementary material for: Constraints to gene flow increase the risk of genome erosion in the Ngorongoro Crater lion population
Source: Commun Biol. 2025 Apr 21;8:640. doi: 10.1038/s42003-025-07986-0 (PMC12012037; doi:10.1038/s42003-025-07986-0)
Supplement: Supplementary file 5 — reporting summary [file 42003_2025_7986_MOESM5_ESM.pdf]

nature portfolio

Corresponding author(s): Nic Dussex
Last updated by author(s): Jan 28, 2025

Reporting Summary

Nature Portfolio wishes to improve the reproducibility of the work that we publish. This form provides structure for consistency and transparency in reporting. For further information on Nature Portfolio policies, see our Editorial Policies and the Editorial Policy Checklist.

Statistics

For all statistical analyses, confirm that the following items are present in the figure legend, table legend, main text, or Methods section.

n/a	Confirmed	
☐	☒	The exact sample size (*n*) for each experimental group/condition, given as a discrete number and unit of measurement
☐	☒	A statement on whether measurements were taken from distinct samples or whether the same sample was measured repeatedly
☐	☒	The statistical test(s) used AND whether they are one- or two-sided<br>*Only common tests should be described solely by name; describe more complex techniques in the Methods section.*
☒	☐	A description of all covariates tested
☒	☐	A description of any assumptions or corrections, such as tests of normality and adjustment for multiple comparisons
☐	☒	A full description of the statistical parameters including central tendency (e.g. means) or other basic estimates (e.g. regression coefficient) AND variation (e.g. standard deviation) or associated estimates of uncertainty (e.g. confidence intervals)
☐	☒	For null hypothesis testing, the test statistic (e.g. *F*, *t*, *r*) with confidence intervals, effect sizes, degrees of freedom and *P* value noted<br>*Give P values as exact values whenever suitable.*
☒	☐	For Bayesian analysis, information on the choice of priors and Markov chain Monte Carlo settings
☒	☐	For hierarchical and complex designs, identification of the appropriate level for tests and full reporting of outcomes
☒	☐	Estimates of effect sizes (e.g. Cohen's *d*, Pearson's *r*), indicating how they were calculated

Our web collection on statistics for biologists contains articles on many of the points above.

Software and code

Policy information about availability of computer code

Data collection	no code was use for data collection
Data analysis	Generode pipeline V1 - https://github.com/NBISweden/GenErode<br>Kutschera, V. E. et al. GenErode: a bioinformatics pipeline to investigate genome erosion in endangered and extinct species. Preprint at https://doi.org/10.1101/2022.03.04.482637.<br><br>GONE V1<br>Santiago, E. et al. Recent Demographic History Inferred by High-Resolution Analysis of Linkage Disequilibrium. Mol. Biol. Evol. 37, 3642–3653 (2020).<br><br>ADMIXTURE v1.3.0<br>Alexander, D. H., Novembre, J. & Lange, K. Fast model-based estimation of ancestry in unrelated individuals. Genome Res. 19, 1655–1664 (2009).<br><br>PPSMC v0.6.5<br>Li, H. & Durbin, R. Fast and accurate long-read alignment with Burrows-Wheeler transform.<br>Bioinformatics 26, 589–595 (2010).<br><br>SMC++ v.1.15.2<br>Terhorst, J., Kamm, J. A. & Song, Y. S. Robust and scalable inference of population history from hundreds of unphased whole genomes. Nature Genetics vol. 49 303–309 Preprint at https://doi.org/10.1038/ng.3748 (2017).

mlRho v2.7
Haubold, B., Pfaffelhuber, P. & Lynch, M. mlRho - a program for estimating the population mutation and recombination rates from shotgun-sequenced diploid genomes. Mol. Ecol. 19 Suppl 1, 277–284 (2010).

PLINK v1.9
Chang, C. C. et al. Second-generation PLINK: rising to the challenge of larger and richer datasets. GigaScience vol. 4 Preprint at https://doi.org/10.1186/s13742-015-0047-8 (2015).

SnpEff v4.3
Cingolani, P. et al. A program for annotating and predicting the effects of single nucleotide polymorphisms, SnpEff: SNPs in the genome of Drosophila melanogaster strain w1118; iso-2; iso-3. Fly 6, 80–92 (2012).

SLiM 4.0
Haller, B. C. & Messer, P. W. SLiM 4: Multispecies Eco-Evolutionary Modeling. Am. Nat. 201, E127–E139 (2023).

Mouse Genome Informatics database (www.informatics.jax.org)

Code for data processing and analysis, and simulation are deposited to Github:
https://github.com/ndussex/Crater_lion_genomics

For manuscripts utilizing custom algorithms or software that are central to the research but not yet described in published literature, software must be made available to editors and reviewers. We strongly encourage code deposition in a community repository (e.g. GitHub). See the Nature Portfolio guidelines for submitting code & software for further information.

Data

Policy information about availability of data

All manuscripts must include a data availability statement. This statement should provide the following information, where applicable:
- Accession codes, unique identifiers, or web links for publicly available datasets
- A description of any restrictions on data availability
- For clinical datasets or third party data, please ensure that the statement adheres to our policy

Resequencing data (ENA BioProject): PRJEB80542, PRJNA611920, PRJNA182708, PRJNA16726, PRJNA854353, PRJNA684344

Research involving human participants, their data, or biological material

Policy information about studies with human participants or human data. See also policy information about sex, gender (identity/presentation), and sexual orientation and race, ethnicity and racism.

Reporting on sex and gender	NA
Reporting on race, ethnicity, or other socially relevant groupings	NA
Population characteristics	NA
Recruitment	NA
Ethics oversight	NA

Note that full information on the approval of the study protocol must also be provided in the manuscript.

Field-specific reporting

Please select the one below that is the best fit for your research. If you are not sure, read the appropriate sections before making your selection.

☒ Life sciences ☐ Behavioural & social sciences ☐ Ecological, evolutionary & environmental sciences

For a reference copy of the document with all sections, see nature.com/documents/nr-reporting-summary-flat.pdf

Life sciences study design

All studies must disclose on these points even when the disclosure is negative.

Sample size	Sample size (n=25), including three outgroup reference genomes (cat, tiger, cheetah)
Data exclusions	No data was excluded
Replication	Findings were not replicated since this is an observational and not an experimental study
Randomization	This is not relevant to our study since this is an observational and not an experimental study
Blinding	Blinding was not possible since this is an observational and not an experimental study

Reporting for specific materials, systems and methods

We require information from authors about some types of materials, experimental systems and methods used in many studies. Here, indicate whether each material, system or method listed is relevant to your study. If you are not sure if a list item applies to your research, read the appropriate section before selecting a response.

Materials & experimental systems

n/a	Involved in the study	
☒	☐	Antibodies
☒	☐	Eukaryotic cell lines
☒	☐	Palaeontology and archaeology
☐	☒	Animals and other organisms
☒	☐	Clinical data
☒	☐	Dual use research of concern
☒	☐	Plants

Methods

n/a	Involved in the study	
☒	☐	ChIP-seq
☒	☐	Flow cytometry
☒	☐	MRI-based neuroimaging

Animals and other research organisms

Policy information about studies involving animals; ARRIVE guidelines recommended for reporting animal research, and Sex and Gender in Research

Laboratory animals	NA
Wild animals	Ear biopsy tissue provided by the Tanzania Wildlife Research Institute (TAWIRI).
Reporting on sex	NA
Field-collected samples	NA
Ethics oversight	The Tanzania Wildlife Research Institute (TAWIRI) approved and performed sampling

Note that full information on the approval of the study protocol must also be provided in the manuscript.

Plants

Seed stocks	NA
Novel plant genotypes	NA
Authentication	NA
